# Genotypic Characterization of Human Respiratory Syncytial Viruses Detected in Mexico Between 2021 and 2024

**DOI:** 10.3390/v17050651

**Published:** 2025-04-30

**Authors:** Nadia Martínez-Marrero, Juan Carlos Muñoz-Escalante, Rosa Maria Wong-Chew, Pedro Torres-González, Miguel Leonardo García-León, Patricia Bautista-Carbajal, Pedro Antonio Martínez-Arce, María del Carmen Espinosa-Sotero, Verónica Tabla-Orozco, Fabian Rojas-Larios, Susana Juárez-Tobías, Ana María González-Ortiz, Ángel Gabriel Alpuche-Solís, Daniel E. Noyola

**Affiliations:** 1 Infectious Diseases Laboratory, Centro de Investigación en Ciencias de la Salud y Biomedicina, Universidad Autónoma de San Luis Potosí, San Luis Potosí 78210, Mexico; nadiamar24@gmail.com (N.M.-M.); carlos.escalante@uaslp.mx (J.C.M.-E.); pedro.torres@uaslp.mx (P.T.-G.); 2 Microbiology Department, Facultad de Medicina, Universidad Autónoma de San Luis Potosí (UASLP), Av. Sierra Leona 550, San Luis Potosi 78210, Mexico; 3 Infectious Diseases Research Laboratory, Research Division, Facultad de Medicina, Universidad Nacional Autónoma de Mexico, Ciudad de Mexico 04510, Mexico; rmwong@unam.mx (R.M.W.-C.);; 4 Hospital Civil de Guadalajara Fray Antonio Alcalde, Guadalajara 44280, Mexico; 5 Hospital General de Mexico “Dr. Eduardo Liceaga”, Ciudad de Mexico 06720, Mexico; 6 Hospital Pediátrico de Coyoacán, Ciudad de Mexico 04100, Mexico; 7 Hospital Regional Universitario de los Servicios de Salud de Colima, Colima 28010, Mexico; 8 Hospital Central “Dr. Ignacio Morones Prieto”, San Luis Potosí 78210, Mexico; 9 Hospital del Niño y la Mujer “Dr. Alberto López Hermosa”, San Luis Potosí 78364, Mexico; anagon71@yahoo.com.mx; 10 Laboratorio de Biología Molecular de Plantas, División de Biología Molecular, Instituto Potosino de Investigación Científica y Tecnológica A.C., San Luis Potosí 78216, Mexico; alpuche@ipicyt.edu.mx

**Keywords:** respiratory syncytial virus, acute respiratory infections, genotype, molecular epidemiology, pneumovirus

## Abstract

Human respiratory syncytial virus (HRSV) is a leading cause of severe respiratory infections among children, older adults, and immunocompromised individuals. The COVID-19 pandemic and the non-pharmacological interventions to mitigate it resulted in significant changes in HRSV epidemiology and seasonality patterns. Worldwide, there was a considerable reduction in the number of HRSV infections during that period, and the impact of those changes on genotype distribution is still not fully understood. In this work, we analyzed the genotypic characteristics of HRSV strains detected between 2021 and 2024 in Mexico with the aim of identifying changes in circulating lineages. HRSV positive samples collected in five states in Mexico were used. The complete viral attachment glycoprotein gene was sequenced, and phylogenetic inference was performed using datasets including all sequences available at GenBank and GISAID until 30 June 2024. We obtained 114 HRSV sequences (63.2% HRSV-A and 36.8% HRSV-B); 19 were from the 2021–2022 season, 53 from 2022–2023, and 42 from 2023–2024. All HRSV-A sequences clustered with sequences from other countries within A.D lineages, including A.D.1, A.D.3, A.D.5.1, and A.D.5.2 lineages. All HRSV-B sequences clustered in the B.D.E.1 lineage with sequences collected between 2020 and 2024. In conclusion, the characterization of HRSV viruses circulating in Mexico during and after the SARS-CoV-2 pandemic and comparison to all available sequences reported to date corroborates that, on a global scale, HRSV-A viruses of several A.D lineages circulate simultaneously, while HRSV-B viruses are restricted to the B.D.E.1 lineage.

## 1. Introduction

Human respiratory syncytial virus (HRSV) is a leading cause of lower respiratory tract infections among all age groups and a significant burden to health care services globally [1]. Children aged 0–5 years, particularly those younger than six months living in low-income and middle-income countries, are disproportionally affected by HRSV [2,3]. Estimates of the mortality burden of HRSV for 2019 indicated 1 in every 50 deaths in children 0–5 years and 1 in every 28 deaths in infants under 6 months that occur worldwide are caused by this virus. In infants and young children, prematurity, chronic respiratory diseases, neurological and neuromuscular disorders, and congenital heart diseases are associated with a more severe course of HRSV infection and the need for hospitalization [4,5]. In addition to the pediatric burden of disease, the World Health Organization recognizes HRSV as an important pathogen in older adults, with infections by this virus leading to an increase in hospitalization rates among those aged 65 years and over, and to increased mortality rates among the elderly and immunocompromised individuals. HRSV is also a nosocomial threat for young infants and immunocompromised and vulnerable individuals [6]. Worldwide, the control of transmission of HRSV remains an unsolved problem.

HRSV is a member of the enveloped negative-sense RNA virus family Pneumoviridae [7]. Considering antigenic reactivity to monoclonal antibodies against viral surface proteins F and G, two antigenic subgroups of HRSV have been identified (A and B), which show genome-wide divergence in nucleotide and amino acid sequence identity [7,8]. Viral strains of both subgroups circulate simultaneously all around the world. HRSV-A infections account for approximately 60% of infections reported globally. However, between 2016 and 2018, HRSV-A and HRSV-B seemed to reach an apparent equilibrium associated with an increase in the overall HRSV-B prevalence [9]. In addition, HRSV exhibits genetic variability within both antigenic subgroups, leading to the identification of many genotypes and lineages. These genotypes may differ in their distribution and prevalence in different regions and at other times [9,10,11]. The attachment glycoprotein G gene has been of particular interest in this context because it is highly variable [12], and partial and complete G gene sequences have been frequently used as the basis for defining HRSV-A and HRSV-B genotypes and lineages [12,13,14]. Molecular epidemiology surveillance of the local HRSV genotypes is essential to understand the evolution, epidemiology, and clinical presentation of the infection by this virus, as well as for timely identification and management of disease outbreaks [13,15,16]. Moreover, HRSV-A and HRSV-B genetic variability analysis is crucial for developing effective prevention and treatment strategies [17,18].

The COVID-19 pandemic was associated with a significant change in seasonality patterns of HRSV, and a considerable reduction in the number of infections caused by this virus occurred worldwide during 2020 [19,20,21]. In this regard, non-pharmacological interventions seem to have been fundamental [22]. The significantly reduced human contact rates, travel restrictions, mask-wearing in public areas, closures of schools and workplaces, and increased hand hygiene led not only to the control of SARS-CoV-2 spread, but also to a reduction in the transmission of respiratory viruses like HRSV [23,24]. Interestingly, once interventions to reduce SARS-CoV-2 transmission were eased, an atypical reemergence of HRSV was described in various countries in both the northern and southern hemispheres [21,25,26]. Several authors described the re-emergence of HRSV following the low incidence in the 2020–2021 season as being caused by previously existing genotypes that have continued circulating in the studied regions [13,16,27,28,29,30]. These studies suggest that the sudden return of HRSV could have resulted from reduced immunity in the general population, rather than from the appearance of novel HRSV variants.

Some studies have reported HRSV circulation in Mexico and have analyzed the viral epidemiology and clinical presentation of infection [31,32,33,34,35]. However, few molecular epidemiology analyses have been conducted regarding the HRSV genotypes that cause infections in Mexico [36,37]. HRSV-A strains of A.3.1 and A.2.1.1 lineages (formerly classified as GA2 and GA5 genotypes) and HRSV B strains of B.D and B.D.1 lineages (formerly classified as BA) were identified between 2004 and 2009 [36]. Starting in 2009, A.D lineage viruses (formerly classified as ON1 genotype) started to circulate in Mexico, and by the 2011–2012 winter season, they comprised almost all HRSV A viruses [37]. Genotypes or lineages of viruses present during and after the COVID-19 pandemic have not been described.

In this study, we conducted a genotypic characterization of HRSV strains detected between 2021 and 2024 in Mexico with the aim of assessing if there are any newly emerging HRSV A or HRSV B lineages.

## 2. Materials and Methods

### 2.1. Samples

Study samples were obtained from surveillance samples collected during the COVID-19 pandemic and from epidemiological studies on respiratory viruses at eight institutions in five states in Mexico. In San Luis Potosí, samples were collected in four institutions: Hospital Central “Dr. Ignacio Morones Prieto”, Hospital del Niño y la Mujer “Dr. Alberto López Hermosa”, Universidad Autónoma de San Luis Potosí (UASLP), and Instituto Potosino de Investigación Científica y Tecnológica, A.C. (IPICYT). Samples from Mexico City were obtained from two hospitals (Hospital Pediátrico de Coyoacán and Hospital General de Mexico “Dr. Eduardo Liceaga”). Michoacán and Jalisco samples were collected in Hospital Civil de Guadalajara “Fray Antonio Alcalde”. In Colima, samples were obtained at Hospital Universitario de los Servicios de Salud del Estado de Colima. The Ethics and Research Committees at participating hospitals (Hospital Civil de Guadalajara 052/20; Hospital General de Mexico “Dr. Eduardo Liceaga” DI/22/505/05/42; Hospital Pediátrico de Coyoacán 101-011-025-21; Hospital Universitario de los Servicios de Salud del Estado de Colima CEI 2022/1/CR/CL/EPI/166; Hospital Central “Dr. Ignacio Morones Prieto” 37-21; and Hospital del Niño y la Mujer-Research and Ethics Committee of the Ministry of Health of the Government of the State of San Luis Potosí SLP/04-2023), as well as the Faculty of Medicine, UNAM (FM/DI/105/2020), approved the corresponding epidemiological studies. Informed consent was obtained from the parents for study participation. Respiratory samples were analyzed for the presence of respiratory viruses using qPCR. Samples collected in Mexico City, Guadalajara, and Colima were stored at −70 °C at the Faculty of Medicine, UNAM; subsequently, viral RNA was extracted from HRSV-positive samples, and cDNA was synthetized and sent to the Microbiology Department, Faculty of Medicine-UASLP, for genotyping. Samples collected in San Luis Potosí were similarly stored and processed at UASLP. All 216 positive samples were subjected to type-specific PCR protocols that amplify the complete coding region of the G gene of HRSV A and HRSV B.

### 2.2. HRSV-A and HRSV-B Genotyping

Genotyping of HRSV was performed by sequencing simple or nested PCR products obtained with sets of primers for the specific amplification of the HRSV-A and HRSV-B complete viral attachment glycoprotein gene (G gene) (Supplementary Table S1). Amplification reactions were executed in 25 μL of final volume with 1X DreamTaqTM Buffer + 2 mM of MgCl2 (Thermo Fisher, Waltham, MA, USA), 0.2 mM of dNTP mix, 1.25 U of DreamTaqTM DNA Polymerase (Thermo Fisher, Waltham, MA, USA), and 0.4 µM of primers HRSV-A-F1/HRSV-A-R1 or HRSV-B-F1/HRSV-B-R1 in the first round of PCR (PCR-1), or 0.2 µM of primers HRSV-A-R2/HRSV-A-F2 or HRSV-B-R2/HRSV-B-F2 for the nested amplification reaction (PCR-2). Considering the detection qPCR Ct values (21–31), 0.5–1.5 μL of cDNA were used as a template for PCR-1, and 0.5–1 μL of PCR-1 amplicon were added for PCR-2.

Thermocycling conditions for PCR-1 were: 95 °C for 3 min, followed by 30 cycles of 30 s at 95 °C, 30 s at 52 °C, 90 s at 72 °C, and finally, 72 °C for 5 min. For PCR-2, the following conditions were used: 95 °C for 3 min, followed by 30 cycles of 30 s at 95 °C, 30 s at 56 °C, 72 °C for 80 s, and lastly, 72 °C for 5 min. All amplifications were created in Applied Biosystems™ Veriti thermocyclers.

For samples in which the results of initial detection by qPCR showed Ct values between 14 and 20, simple PCR with primers for PCR-2 was performed; the quantity of cDNA used in those cases ranged from 1–2.5 μL, and 35 cycles of amplification were used.

Oligonucleotide pairs for PCR-2 generate bands of approximately 1300 bp in both HRSV-A and HRSV-B, which makes it possible to observe PCR results using 1% agarose gels. Five to ten microliters of PCR-2 amplicon were purified with ExoSAP-ITTM (Applied Biosystems, Foster City, CA, USA), as suggested by the manufacturer, and submitted to Sanger Sequencing using the BigDye Terminator Sequencing Kit v1.1 and an ABI 3500 Genetic Analyzer (both Applied Biosystems, Foster City, CA, USA), in LANBAMA-IPICYT (San Luis Potosí, Mexico). Sequences were manually edited with BioEdit [38].

### 2.3. Global HRSV Sequences

HRSV sequences available in GenBank and GISAID until 30 July 2024 were downloaded. For GenBank sequences taxonomy (txid208893 for HRSV-A and txid208895 for HRSV-B) and sequence length filters (>800 nucleotide length) were used to download each dataset; for GISAID, the totality of HRSV-A and HRSV-B sequences deposited until July 30, 2024 were downloaded separately. HRSV-A and HRSV-B sequences were independently aligned using MAFFT version 7.450 [39]. Inspection and correction of alignment artifacts were manually performed with BioEdit v.7.2.5. Alignments were trimmed to span the complete G gene using HRSV-A (NC_038235) and HRSV-B (NC_001781) as reference sequences. Sequences with ambiguities of more than 5% and sequences spanning less than the complete G gene were removed from the alignments. To avoid redundancy during phylogenetic analysis, identical sequences were removed from the final alignment using ElimDupes (https://www.hiv.lanl.gov/content/sequence/elimdupesv2/elimdupes.html accessed on 7 October 2024 and 23 October 2024).

Sample information (including the collection date and geographic location) from sequences comprehending the final HRSV-A and HRSV-B datasets was registered. Lineages reference datasets proposed by Goya et al. were integrated into the respective HRSV-A and HRSV-B final alignments for subsequent phylogenetic analysis and lineage assignation [40].

### 2.4. Recombination Analysis

A recombination detection analysis was performed using RDP5 [41] to maximize the accuracy of the phylogenetic analysis. Possible recombination events were evaluated using RDP, GENECONV, Chimaera, Max-Chi, SiScan, and 3Seq algorithms. True recombination events were defined as those detected for at least five different algorithms using RDP5.

### 2.5. Phylogenetic Analysis

Phylogenetic inference for HRSV-A and HRSV-B sequence datasets was performed with IQ-TREE v2.2.2.6 (http://www.iqtree.org) using the Maximum-likelihood method under the best fitting substitution model, as inferred by the ModelFinder tool (GTR+F+I+R6 for both the HRSV-A and HRSV-B datasets), and integrated into IQ-TREE v2.2.2.6. Trees were visualized using FigTree software v.1.4.4 (http://tree.bio.ed.ac.uk/software/figtree/). Assessment of branch supports was determined by SH-aLRT [42] and UFBoot2 with 1000 and 10,000 replicates, respectively [43]. Monophyletic clades were considered statistically supported when the SH-aLRT value was >80% and the UFBoot2 value was >90%.

### 2.6. Lineage Assignment

The phylogenetic trees were rooted against A.1 (for the HRSV-A dataset) or B.1 (for the HRSV-B dataset) clades, and lineages were assigned to every sequence by associating them with the lineage reference sequences, taking clade statistical support into account, as previously described [40]. Lineage assignation information was added to the geo-temporal information data for subsequent analysis.

### 2.7. Analysis of Amino Acid Sequences and Amino Acid-Based Lineage Definition

The amino acid sequences were deduced for HRSV-A and HRSV-B and were compared to the reference sequences previously used to describe lineage-defining amino acids: HRSV/A/England/397/2017 (lineage A.D.2.2.1, GenBank accession no. PP109421.1, GISAID EpiRSV accession no. EPI_ISL_412866) or HRSV/B/AUS/VIC-RCH056/2019 (lineage B.D.4.1.1, GenBank accession no. OP975389.1, GISAID EpiRSV accession no. EPI_ISL_1653999) [40].

## 3. Results

**Figure 1:**
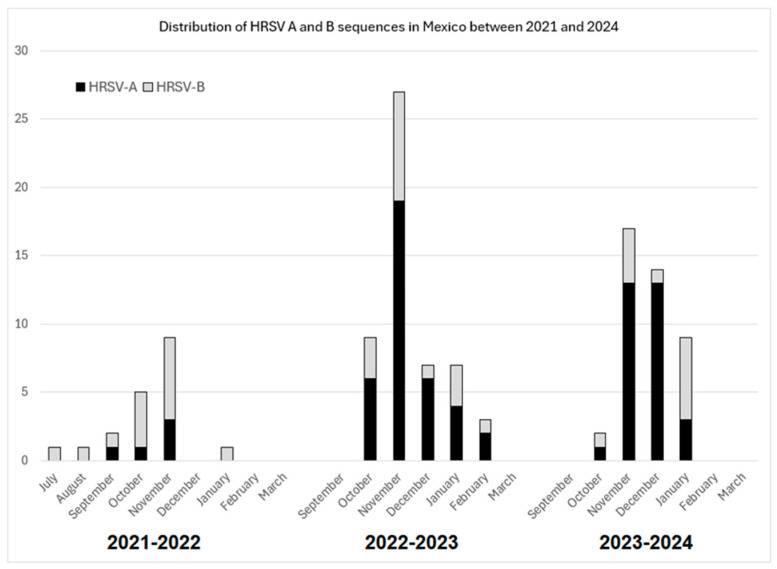
Monthly numbers of HRSV-A and HRSV-B sequences from Mexican patients during the 2021–2022, 2022–2023, and 2023–2024 winter seasons.

In total, we obtained 114 HRSV sequences from samples collected during 2021 (n = 18), 2022 (n = 44), 2023 (n = 43), and 2024 (n = 9). Samples were collected from patients from San Luis Potosí (n = 74), Jalisco (n = 23), Mexico City (n = 12), Colima (n = 4), and Michoacán (n = 1). Considering the seasonal pattern of HRSV circulation in Mexico (northern hemisphere), 19 samples corresponded to the 2021–2022 season, 53 to the 2022–2023 season, and 42 to the 2023–2024 season. Overall, 72 (63.2%) were HRSV-A and 42 (36.8%) were HRSV-B. The numbers of sequences obtained during each month and winter season are shown in Figure 1. These sequences were obtained from a group of 216 patients, in whom HRSV was detected, among 1316 patients who were tested at the various study sites (Table 1). The sequences were submitted to GenBank with accession numbers PQ834837–PQ834950.

Most patients included in the study (110, 96.5%) were children (<18 years of age) and were hospitalized due to HRSV infection (97, 85.1%). The most frequent diagnoses were pneumonia (78, 68.4%), bronchiolitis (18, 15.8%), and unspecified acute respiratory infection (14, 12.3%). Most patients were previously healthy, and 23 (20.2%) had one or more underlying conditions; the most frequent underlying conditions were preterm birth (14, 12.3%), asthma (5, 4.4%), and congenital heart disease (5, 4.4%). The patient characteristics according to HRSV type and lineage are shown in Supplementary Table S2.

### 3.1. Recombination Analysis

No evidence of recombination events was found in the Mexican HRSV-A or HRSV-B sequence alignments (Supplementary Material Figures S1 and S2).

### 3.2. Global HRSV Sequences

A total of 9226 HRSV-A and 7836 HRSV-B sequences were downloaded and processed from GenBank, while 37,706 HRSV-A and 20,457 HRSV-B sequences were downloaded and processed from GISAID. After the curation of the datasets, 5181 HRSV-A and 3917 HRSV-B sequences from GenBank and 10,652 HRSV-A sequences and 8297 HRSV-B sequences from GISAID were included in the analysis. Only 11 HRSV-A and 15 HRSV-B sequences from Mexico containing the full-length G gene were identified in GenBank and GISAID based on our inclusion and exclusion criteria.

### 3.3. Phylogenetic Inference and Lineage Assignment

All obtained HRSV-A sequences were 966 nucleotides in length from ATG to TGA (as stop codon), corresponding to the entire coding sequence of the G protein. In the case of HRSV-B, the sequences present a length of 933 nucleotides from ATG to the triplet TAA as stop codon, also corresponding to the G protein ORF. The 114 obtained sequences (both HRSV-A and B) had a partial duplication in the G gene.

**Figure 2:**
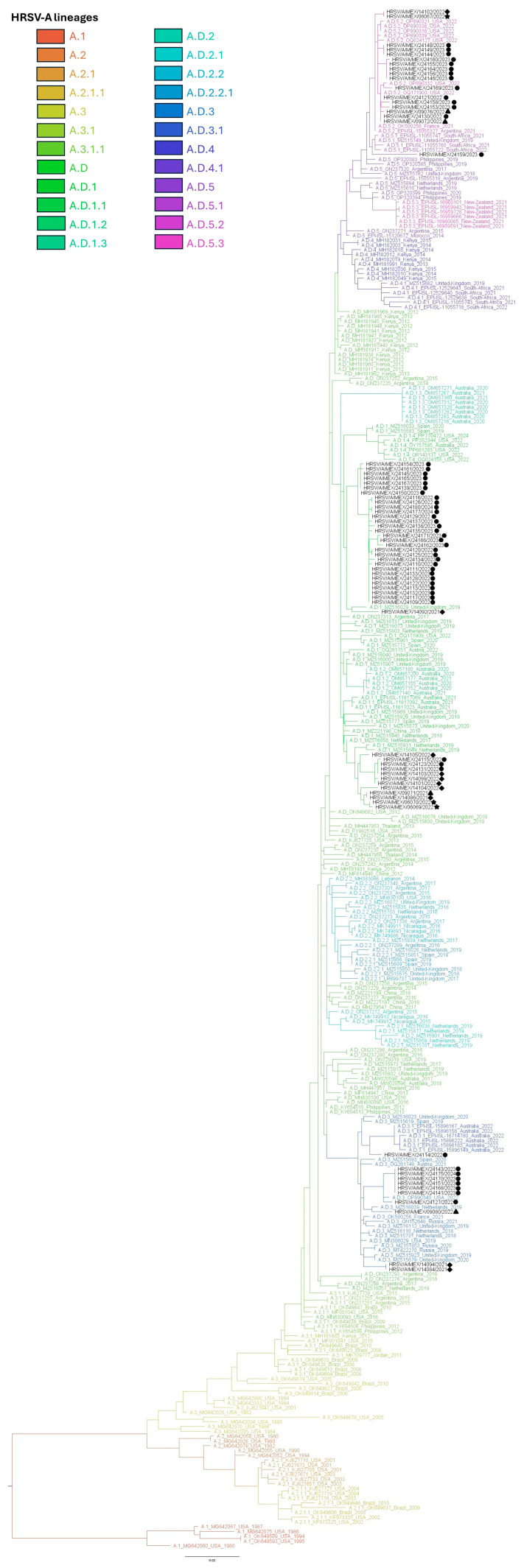
HRSV-A Maximum-likelihood phylogenetic analysis of complete G gene sequences from this study and reference sequences for each lineage. Sequences from Mexico are shown in black. Markers at the tips of sequences indicate the state of origin for each sequence (circle, San Luis Potosí; triangle, Mexico City; diamond, Jalisco; star, Colima).

Phylogenetic inference showed that all HRSV-A sequences clustered within A.D lineages (Figure 2 and Supplementary Figure S3). Sequences were distributed in clades along A.D.1, A.D.3, A.D.5.1, and A.D.5.2 lineages and clustered with sequences from samples collected between 2020 and 2024 in other countries (Table 2 and Supplementary Figure S4).

**Figure 3:**
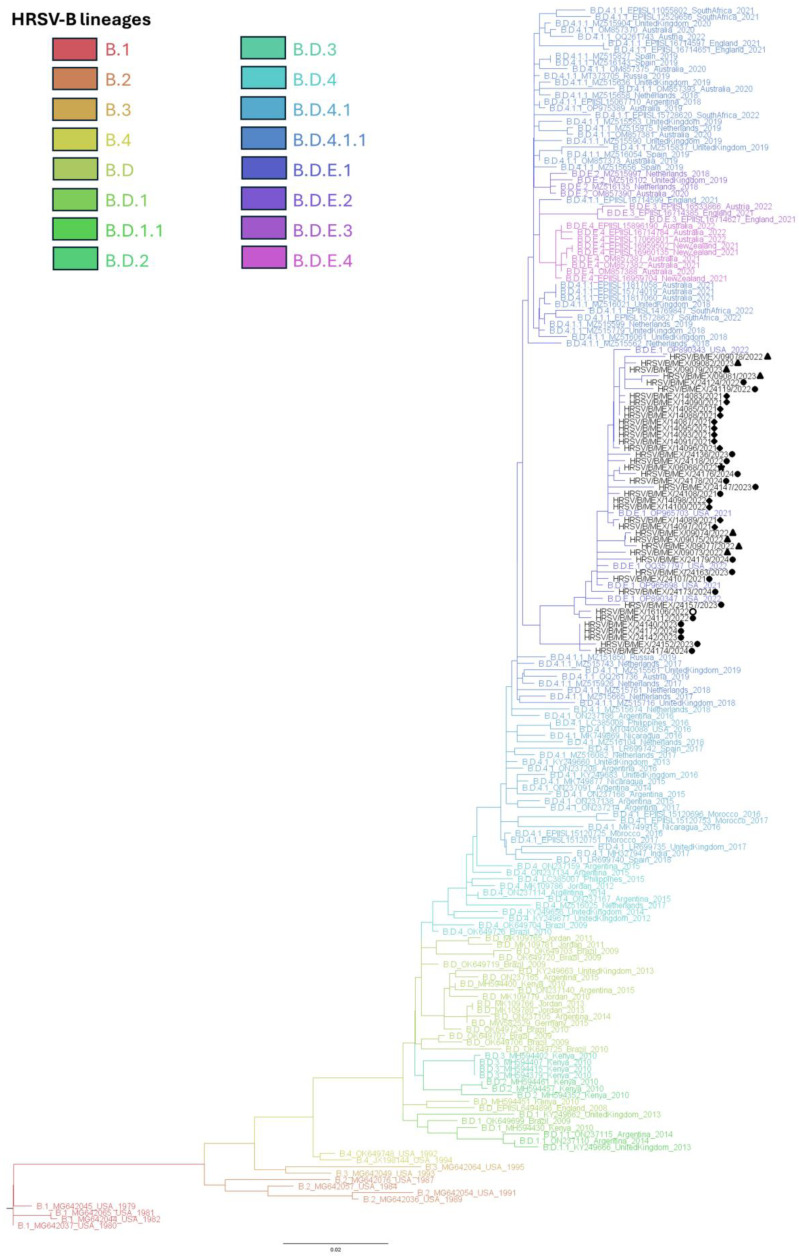
HRSV-B Maximum-likelihood phylogenetic analysis of complete G gene sequences from this study and reference sequences for each lineage. Sequences from Mexico are shown in black. Markers at the tips of sequences indicate the state of origin for each sequence (circle, San Luis Potosí; triangle, Mexico City; diamond, Jalisco; star, Colima; empty circle, Michoacán).

In contrast, for HRSV-B, phylogenetic inference revealed that all HRSV-B sequences clustered within a single lineage (B.D.E.1) (Figure 3 and Supplementary Figure S5), along with most sequences from samples collected between 2020 and 2024 worldwide (Table 3 and Supplementary Figure S6).

**Figure 4:**
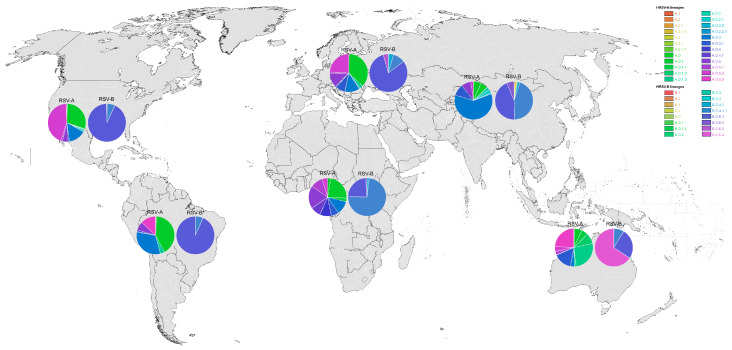
HRSV-A and HRSV-B lineage distribution of sequences reported between 2021 and 2024 in each continent.

To better understand the current global HRSV lineage distribution, we analyzed the proportion of sequences of each lineage in each continent (and those found in this study in Mexico). The distribution of HRSVA lineages in Mexico follows a pattern similar to that observed in Africa, Asia, Europe, North America, and South America, where the predominant lineages were A.D.1, A.D.3, and A.D.5.2 (Supplementary Table S3 and Figure 4). In contrast, sequences from Oceania were predominantly classified as lineages A.D.1.3, A.D.3.1, and A.D.5.3. Regarding RSVB sequences, lineage B.D.E.1 was the most prevalent in Mexico, as well as in Europe, North America, and South America. In Asia, both B.D.4.1.1 and B.D.E.1 were dominant, while in Africa, B.D.4.1 was the most frequently assigned lineage (Supplementary Table S3 and Figure 4). Unlike all other regions, Oceania exhibited a marked predominance of the B.D.E.4 lineage, which appears to be geographically restricted to that continent.

### 3.4. Analysis of Amino Acid Sequences and Amino Acid-Based Lineage Definition

**Figure 5:**
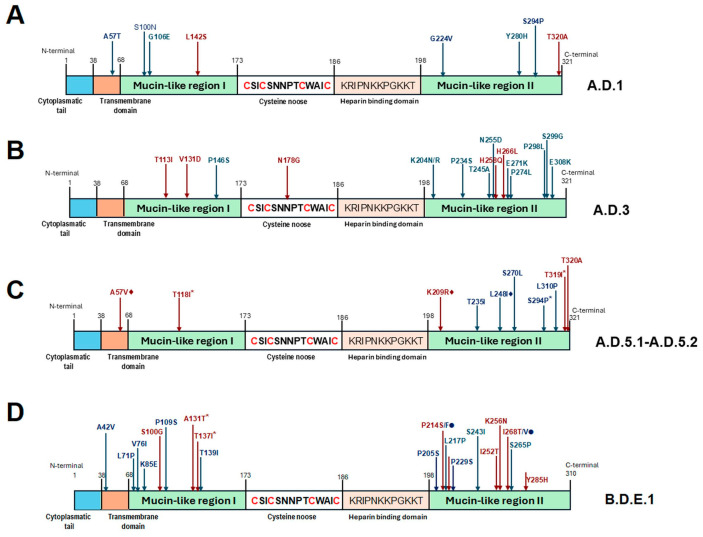
Distribution of amino acid changes of the G protein present in (**A**) A.D.1, (**B**) A.D.3, (**C**) A.D.5.1/A.D.5.2, and (**D**) B.D.E.1 HRSV lineages. In panel (**C**), diamonds (⧫) indicate changes present in both A.D.5.1 and A.D.5.2, while asterisks (*) indicate changes present only in A.D.5.1. In panel (**D**), circles (●) indicate changes not reported by Goya et al. [40] and asterisks (*) indicate changes present in B.D.4.1.1.

Deduced amino acid sequences of 322 and 311 residues were obtained for all HRSV-A and HRSV-B sequences, respectively. HRSV-A sequences had conserved TGA triplet as stop codons at position 322 and exhibited amino acid substitutions in comparison with the above-mentioned reference sequence. In 66/72 (91.6%) sequences, it was possible to recognize the lineage-defining amino acids proposed by Goya et al. in 2024 for the G gene [40]. In six sequences assigned to A.D.1 lineage, the parental threonine is conserved at position 320; they represent 13.9% of all A.D.1 sequences. The amino acid-based lineage analysis for HRSV-A is summarized in Figure 5 and Supplementary Table S4.

Of note, in the case of HRSV-A, 23/43 (53.4%) sequences defined as lineage A.D.1 also shared A57T + S100N + G224V + S294P changes. Only 150/3268 (4.2%) sequences in the dataset displayed these four changes; they were mainly reported in Spain (2023–2024) and the United States (2022–2024). Sequences with those changes represent 20.8% (90/433) and 5.2% (46/884) of the A.D.1 sequences from Spain and the United States used in the analysis, respectively. It is also important to highlight that the combination of two additional changes, G106E + Y280H, also appeared in 8/43 (18.6%) of the Mexican sequences assigned as A.D.1. In the A.D.1 dataset, only 154/3268 (4.7%) sequences show these two changes, with most of them reported in the United States representing 12.1% (107/884) of the total A.D.1 sequences from the United States in the dataset. In contrast to this work, these two amino acid changes are present in sequences from viruses detected in the United States between 2019 and 2024, but the highest percentage corresponds to 2022 (58.8%, 63/107).

Also, 6/11 (54.5%) sequences defined as A.D.3 shared, in addition to the lineage-defining amino acid changes, the following changes: P146S + K204R + T245A + P298L + S299G. This combination of changes was detected in only 4/1770 (0.2%) A.D.3 sequences included in the dataset used in the analyses. The four sequences corresponded to 12.9% (4/308) of the A.D.3 sequences from the United States available in the dataset. We also identified additional amino acid substitutions in 15/17 (88.2%) sequences assigned to A.D.5.2 lineage: T235I + L248I. There are 703/1619 (43.4%) A.D.5.2 sequences with these two changes in the dataset. Most of them correspond to samples collected in the United States between 2021 and 2024. Interestingly, these sequences represent 89.3% (638/714) of the total United States sequences classified as A.D.5.2 used in this work. Twelve of the fifteen sequences from Mexico with the previously described amino acid substitutions also presented an L310P change. In the study dataset, we detected this change in 1583/1634 (96.8%) sequences, principally in 704/714 (98.5%) sequences corresponding to the United States, 291/300 (97%) to Spain, and 152/157 (96.8%) to Great Britain, and obtained from samples collected from 2021 to 2024.

In the case of HRSV-B, sequences had conserved TAA triplets as stop codons at position 311 and also exhibited some amino acid substitutions in comparison with the above-mentioned reference sequence. In 33/42 (78.6%) sequences, it was possible recognize the lineage-defining amino acids proposed by Goya et al. in 2024 for the G gene [40]. In nine sequences, not all lineage-defining amino acid changes were present, or there were additional amino acid substitutions. The amino acid-based lineages analysis for HRSV-B is shown in Figure 5 and Supplementary Table S5.

Of note, 15/42 (35.7%) HRSV-B sequences defined as lineage B.D.E.1 had the T139I substitution in addition to the lineage-defining changes, and 11/42 (26.2%) sequences showed an S265P change. In the dataset employed for HRSV-B analyses, the T139I change appears in 228/3417 (6.7%) sequences. Most of the sequences showing this change are from the United States, were obtained from samples collected between 2021 and 2024, and represent 9.3% (98/1055) of the total of B.D.E.1 sequences reported from this country. A T139I change also appears in sequences from Spain and Great Britain from 2022 and 2023, representing 8.3% (41/491) and 4.2% (25/596) of their total of sequences in the dataset. The S265P change was observed in 519/3417 (15.2%) sequences, mainly from Germany in the years 2021 and 2022, the United States between 2019 and 2024, and Spain from 2021 to 2023. Interestingly, this change is present in 65.5% (154/235) of the total B.D.E.1 sequences from Germany, but in only 11.5% (121/1055) and 18.5% (91/491) of sequences from North America and Spain, respectively.

## 4. Discussion

Molecular epidemiology studies are relevant to understanding viral evolution and characterizing currently circulating viruses to assess the anticipated effectiveness of specific preventive interventions. The COVID-19 pandemic had a major effect on HRSV epidemiology with a reduction of infections during the 2020–2021 winter season, an increase in hospitalizations during subsequent seasons, and changes in seasonality [19,20,21,22,23,24,25,26]. Whether these changes are a result of SARS-CoV-2 circulation, non-pharmacological interventions to control the pandemic, or the emergence of new HRSV lineages has not been completely defined. Therefore, analysis of HRSV genotypes and lineages circulating worldwide is highly relevant.

Studies from the United States [28,29,44], Germany [16], and other countries have characterized HRSV-A and HRSV-B viruses circulating during the pandemic [14,27,45]. Overall, identified viruses belong to lineages circulating at the time of the onset of the SARS-CoV-2 pandemic. A notable finding in these reports is the difference in the distribution of HRSV-A and HRSV-B sequences; while HRSV-A viruses show more variability, clustering along several clades within A.D lineages, HRSV-B diversity has become more restricted. Our results show that, in Mexico, the same pattern of HRSV circulation has been present over the past few years: HRSV-A viruses identified between 2021 and 2024 are distributed along four different clades, A.D.1, A.D.3, A.D.5.1, and A.D.5.2, while all HRSV-B sequences cluster together in the B.D.E.1 lineage. Furthermore, these lineages encompass the majority of HRSV sequences reported worldwide between 2021 and 2024. While the aforementioned lineages have been detected in all continents, some regional variations are observed. Of particular interest is the lineage distribution observed in Oceania, where the most frequently detected lineages were A.D.1.3, A.D.5.3, and B.D.E.4, all of which have rarely been identified in other continents. It will be of interest to observe whether this pattern will persist or change in the upcoming years.

By analyzing the deduced amino acid sequences, we could identify the presence of the amino acid markers described by Goya et al. as lineage identifiers in most HRSV sequences in this work. However, some of these amino acid markers were not present in some sequences. Nevertheless, we found a high concordance between the lineages assigned through phylogenetical analysis and those inferred based on site-specific amino acid substitutions. In addition, based on the considerably high number of A.D.5.2 sequences in the dataset (with representation from several geographical regions and from several years), we considered the L310P amino acid change could probably be considered another A.D.5.2 linage definition marker.

Of note, the distribution of HRSV-A sequences in different lineages in Mexico was not determined by temporal or geographical characteristics: Sequences from different years or states included in our study were present within each of the above-described lineages. In addition, Mexican sequences clustered with sequences from other countries. As such, our results contribute to confirming that a diverse array of viruses within the HRSV-A group cocirculate globally. In contrast, HRSV-B group viruses have shown a bottleneck effect, with a reduction in viral variability. A single lineage (B.D.E.1) is responsible for most infections occurring worldwide in recent years. Before the SARS-CoV-2 pandemic, a predominance of this lineage had been observed; however, cocirculation with other HRSV-B lineages was present at that time [9].

A limitation of our study is that analysis was restricted to the G gene. However, while the use of complete genomes is currently suggested for assignment analysis by several authors, prior studies have shown that analysis of the complete G gene region allows for accurate genotyping [46,47].

Although we could not obtain sequences from all HRSV-positive samples, likely due to low viral load in some patients, our data constitute a substantial contribution to the molecular epidemiology of HRSV in Mexico, since samples from several states were included, and we obtained 114 complete G gene sequences. In contrast, only 24 HRSV G gene sequences were available at GenBank or GISAID from samples collected before 2021.

## 5. Conclusions

The genotypic characterization provided in this work allowed us to obtain knowledge about the molecular characteristics and phylogeny of viruses circulating in Mexico during the analyzed period. This information will serve as a comparative basis for new molecular epidemiology studies of HRSV in Mexico at the post-COVID-19 stage.

Our results show that HRSV-A and HRSV-B strains circulating in Mexico belong to previously described genotypes, and no newly emerging lineages were identified. HRSV sequences from Mexico cluster with viral sequences currently in circulation in other countries on a global scale, with diverse A.D lineages circulating simultaneously (including A.D.1, A.D.3, and A.D.5.2), while HRSV-B viruses are restricted only to the B.D.E.1 lineage. Of interest, the current genotype distribution pattern in Oceania differs from the patterns of other continents. This behavior could be the result of different evolutive responses to external pressures, such as those resulting from SARS-CoV-2 circulation and changes in social interactions established to reduce the impact of this virus. Understanding of the mechanisms leading to these differences in evolutionary behavior could help assess the effects of preventive interventions.

## Figures and Tables

**Table 1:** Numbers of samples tested for HRSV and numbers of positive samples and sequences obtained.

Sample Collection Site	Number of Samples in Which HRSV Was Tested	HRSV-Positive Diagnosis	HRSV-A Specific PCR Positive	HRSV-A Sequences	HRSV-B Specific PCR Positive	HRSV-B Sequences
Hospital General de Colima	41	6	3	3	1	1
Hospital General de Mexico	92	16	3	2	6	6
Hospital Pediátrico de Coyoacán	146	28	2	2	2	2
Hospital Civil de Guadalajara	161	35	10	10	15	14
Hospital Central-SLP	152	35	14	13	2	2
Hospital del Niño y la Mujer	390	160	34	30	14	12
IPICYT	50	5	0	0	1	1
UASLP	284	28	12	12	4	4
Total	1316	216	78	72	45	42

**Table 2:** Distribution of HRSV-A sequences according to lineage, year of sample collection, and geographic location (Global and Mexico).

Lineage	1956–2019	1956–2019	2020	2020	2021	2021	2022	2022	2023	2023	2024	2024
	Global *	Mexico **	Global	Mexico	Global	Mexico	Global	Mexico	Global	Mexico	Global	Mexico
A.1	21											
A.2	30											
A.2.1	323											
A.2.1.1	99	1										
A.3	81								2			
A.3.1	794	1										
A.3.1.1	343											
A.D	2776	5	119		23		26		8		1	
A.D.1	1114		358		242	3	459	22	786	16	303	2
A.D.1.1	43		26		51		19		1		1	
A.D.1.2			141		80							
A.D.1.3	3		266		215		25		1			
A.D.1.4	3		5		17		24		64		10	
A.D.2	35		5		14		27		14		2	
A.D.2.1	54		19		1		9		2		1	
A.D.2.2	667		87	2	3							
A.D.2.2.1	124		6				2					
A.D.3	520		207		329	2	307	3	277	5	128	1
A.D.3.1	11		2		94		223		131		17	
A.D.4	276		2		15		12		44		6	
A.D.4.1	131		7		31		8		3			
A.D.5	319		76		218		142		184		47	
A.D.5.1	10				8		61		69	1	10	
A.D.5.2	6		3		256		784	6	423	11	143	
A.D.5.3	6		6		219		7		5			
Total	7789	7	1335	2	1816	5	2135	31	2014	33	669	3

* Global sequences do not include sequences from Mexico, as these are included in a separate column for comparison. ** Sequences from Mexico include nine sequences reported in GenBank or GISAID and collected before 2021 (shown in black numbers), and 72 sequences from 2021 to 2024 obtained in our study (shown in red numbers). Global genotype distribution for each period/year are color coded from the most frequent lineage (red), intermediate frequency lineages (orange and yellow), to low frequency lineages (green).

**Table 3:** Distribution of HRSV-B sequences according to lineage, year of sample collection, and geographic location (Global and Mexico).

Lineage	1956–2019	1956–2019	2020	2020	2021	2021	2022	2022	2023	2023	2024	2024
	Global *	Mexico **	Global	Mexico	Global	Mexico	Global	Mexico	Global	Mexico	Global	Mexico
B.1	32											
B.2	63											
B.3	85											
B.4	363	3										
B.D	1042	1							1			
B.D.1	147											
B.D.1.1	22											
B.D.2	47											
B.D.3	99											
B.D.4	524											
B.D.4.1	837		14	2	68		43					
B.D.4.1.1	3311		408	9	285		211		118		15	
B.D.E.1	15		6		778	13	1448	13	977	10	183	6
B.D.E.2	79		29		15		5		5			
B.D.E.3			4		83		10		4			
B.D.E.4	3		23		304		309		8			
Not assignable	76				2							
Total	6745	4	484	11	1535	13	2026	13	1113	10	198	6

* Global sequences do not include sequences from Mexico, as these are included in a separate column for comparison. ** Sequences from Mexico include fifteen sequences reported in GenBank or GISAID and collected before 2021 (shown in black numbers), and 42 sequences from 2021 to 2024 obtained in our study (shown in red numbers). Global genotype distribution for each period/year are color coded from the most frequent lineage (red), intermediate frequency lineages (orange and yellow), to low frequency lineages (green).

## Data Availability

Data used for this study are available at public repositories including GenBank and GISAID. Sequences reported in this study have been submitted to GenBank with accession numbers PQ834837–PQ834950.

## Supplementary Materials

The following supporting information can be downloaded at https://www.mdpi.com/article/10.3390/v17050651/s1: Supplementary Figure S1. Results of recombination analysis of Mexican HRSV-A sequence alignments made with RDP v5.64; Supplementary Figure S2. Results of recombination analysis of Mexican HRSV-B sequence alignments made with RDP v5.64; Supplementary Figure S3. HRSV-A Maximum-likelihood phylogenetic analysis of complete G gene sequences from this study and all sequences available at GenBank until 30 July 2024; Supplementary Figure S4. Global distribution of HRSV-A lineages from 2020 to 2024; Supplementary Figure S5. HRSV-B Maximum-likelihood phylogenetic analysis of complete G gene sequences from this study and all sequences available at GenBank until 30 July 2024; Supplementary Figure S6. Global distribution of HRSV-B lineages from 2020 to 2024; Supplementary Table S1. Primers used for amplification of complete HRSV G gene; Supplementary Table S2. Main characteristics of HRSV positive patients included in the study; Supplementary Table S3. HRSV lineage distribution in each continent and Mexico for sequences reported between 2021 and 2024; Supplementary Table S4. Lineage specific amino acid and other changes present in HRSV-A sequences from Mexico; and Supplementary Table S5. Lineage specific amino acid and other changes present in HRSV-B sequences from Mexico.
