# Supplementary material for: Genotypic Characterization of Human Respiratory Syncytial Viruses Detected in Mexico Between 2021 and 2024
Source: Viruses. 2025 Apr 30;17(5):651. doi: 10.3390/v17050651 (PMC12115737; doi:10.3390/v17050651)
Supplement: Supplementary file 1 [file viruses-17-00651-s001.zip › viruses-3570705-supplementary.pdf]

Genotypic Characterization of Human Respiratory Syncytial Viruses Detected in Mexico Between 2021 and 2024

Supplementary Material

Supplementary Figure S1. Results of recombination analysis of Mexican HRSV -A sequence alignments made with RDP v5.64

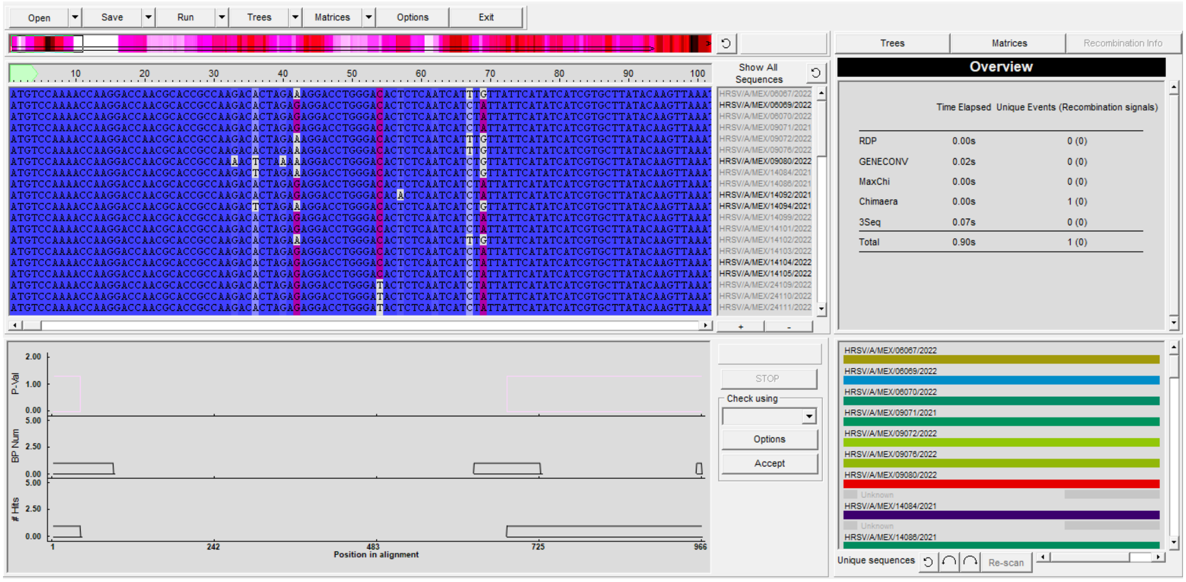

Supplementary Figure S2. Results of recombination analysis of Mexican HRSV-B sequence alignments made with RDP v5.64

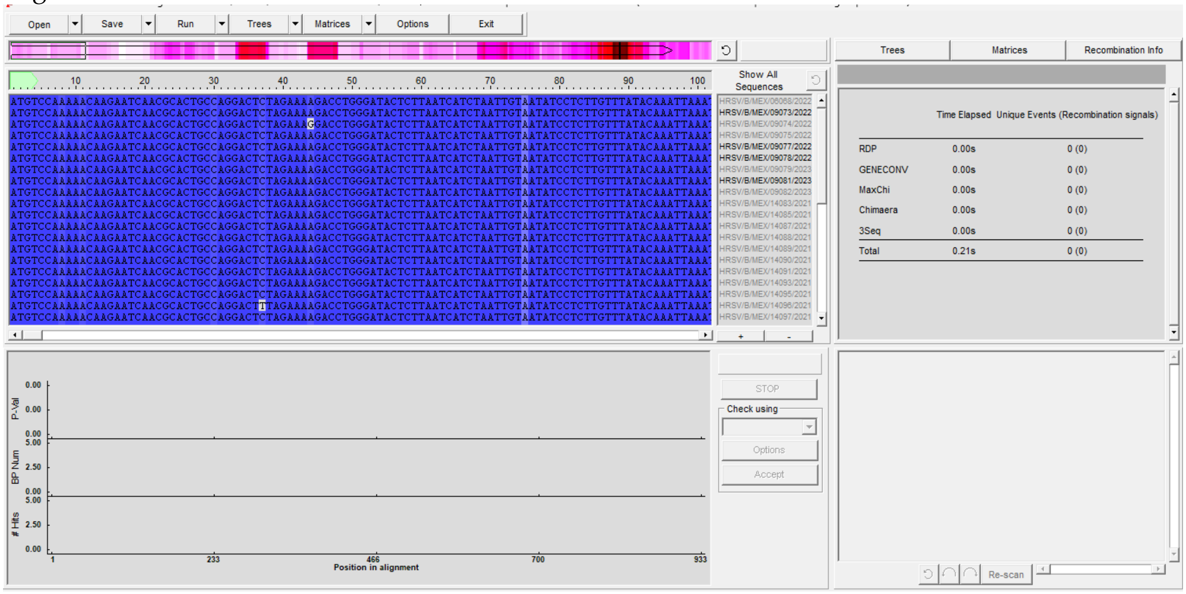

Supplementary Figure S3. HRSV-A Maximum-likelihood phylogenetic analysis of complete G gene sequences from this study and all sequences available at GenBank until 30 July 2024

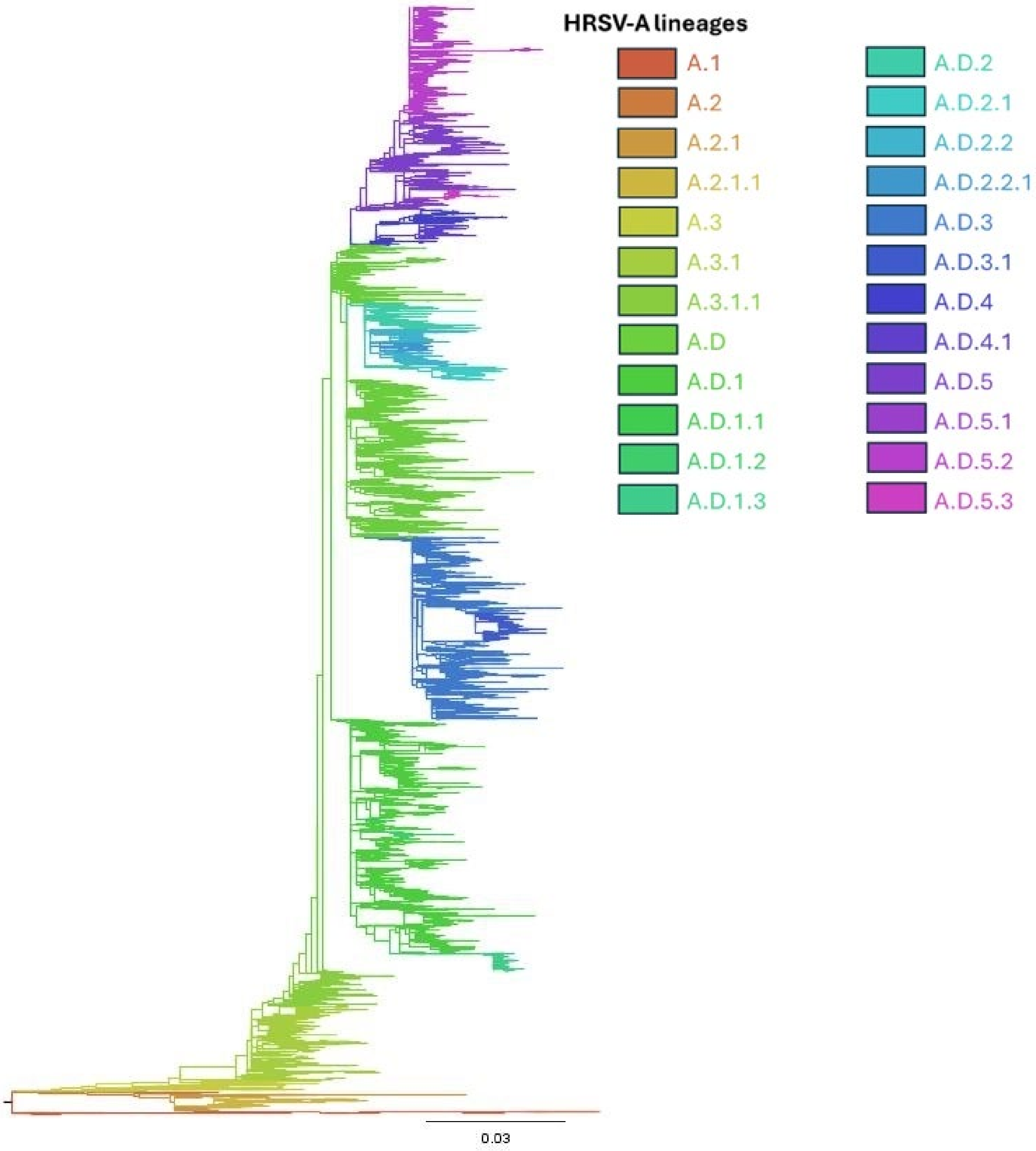

Supplementary Figure S4. Global distribution of HRSV-A lineages from 2020 to 2024.

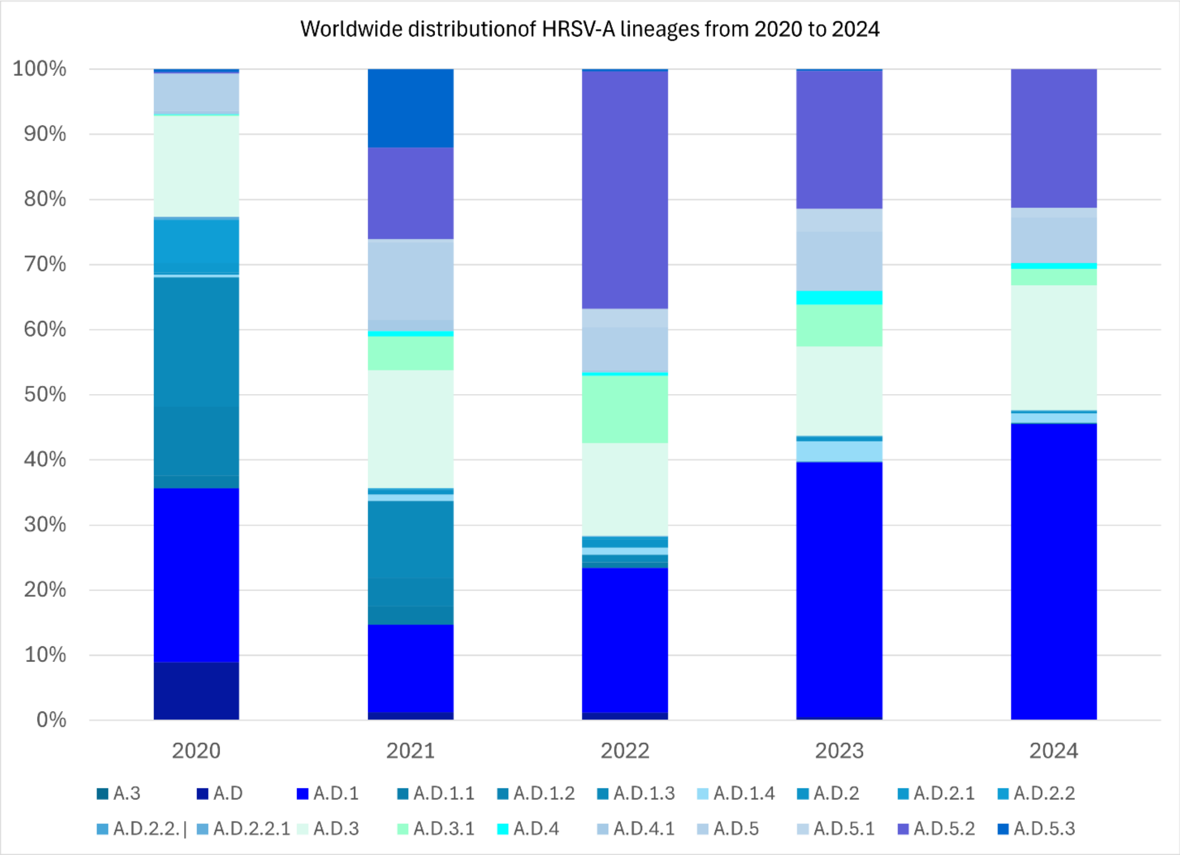

Supplementary Figure S5. HRSVB Maximum-likelihood phylogenetic analysis of complete G gene sequences from this study and all sequences available at GenBank until 30 July 2024

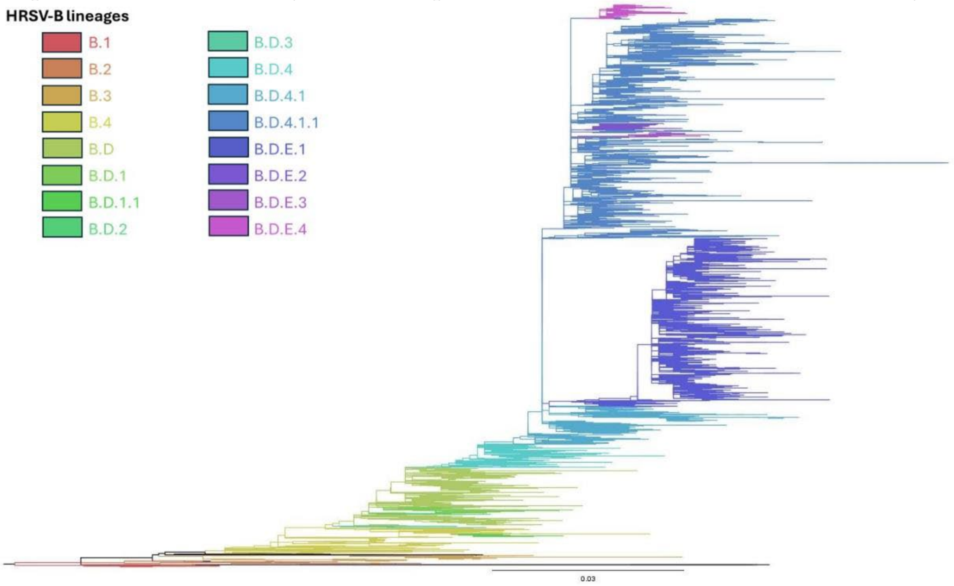

Supplementary Figure S6. Global distribution of HRSV-B lineages from 2020 to 2024

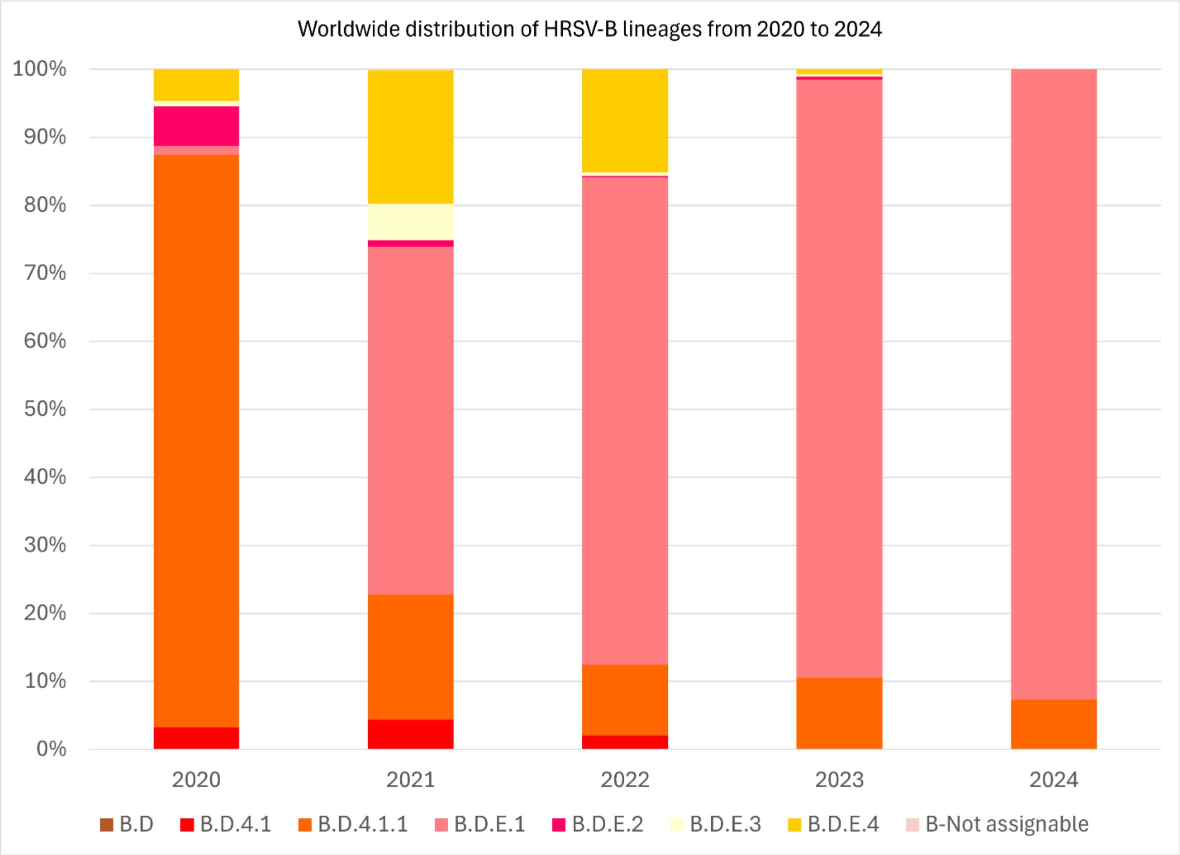

Supplementary Table S1. Primers used for amplification of complete HRSV G gene.

Primers name	Primers sequence (5´- 3´)	References
HRSV-A-F1	TCAAGCAAATTCTGGCCTTA	[1]
HRSV-A-R1_mod	CAACTGCAATTCTGTTACAGCA	[2]
HRSV-A-F2	CCTTTGAGCTACCAAGAGCTC	[1]
HRSV-A-R2	GAGTGTGACTGCAGCAAGGA	[1]
HRSV-B-F1	ACAAGCAAATTTTGGCCCTA	[3]
HRSV-B-R1	TAACTGTAATTCTGTTACTGCA	[3]
HRSV-B-F2	CTCTTGAACAAGGACAGATGTATC	[3]
HRSV-B-R2	CAATGCATTAATAGCAAGAG	[3]

Supplementary Table S2. Main characteristics of HRSV positive patients included in the study.

Patient characteristics	HRSV A Total n=72	(%)	A.D.1 n=43	(%)	A.D.3 n=11	(%)	A.D.5 n=18	(%)	HRSV B B.D.E.1 n=42	(%)	Total n=114	(%)
**Age group**												
Pediatric (< 18 years)	70	97.2	42	97.7	11	100	17	94.4	40	95.2	110	96.5
Adult (18 years or older)	2	2.8	1	2.3			1	5.6	2	4.8	4	3.5
**Diagnosis**												
Pneumonia	46	63.9	27	62.8	8	72.7	11	61.1	32	76.2	78	68.4
Bronchiolitis	14	19.4	6	14.0	3	27.3	5	27.8	4	9.5	18	15.8
Asthma exacerbation									1	2.4	1	0.9
Influenza like illness									1	2.4	1	0.9
Unspecified acute respiratory infection	10	13.9	9	20.9			1	5.6	4	9.5	14	12.3
NA*	2	2.8	1	2.3			1	5.6			2	1.8
**Hospitalization**												
Yes	60	83.3	33	76.7	11	100	16	88.9	37	88.1	97	85.1
No	12	16.7	10	23.3			2	11.1	5	11.9	17	14.9
**Immunosuppression**												
Yes	2	2.8	1	2.3			1	5.6	1	2.4	3	2.6
No	58	80.6	32	74.4	11	100.0	15	83.3	37	88.1	95	83.3
NA	12	16.7	10	23.3			2	11.1	4	9.5	16	14.0
**Underlying conditions**												
Underlying condition	17	23.6	9	20.9	5	45.5	3	16.7	6	14.3	23	20.2
None	43	59.7	24	55.8	6	54.5	13	72.2	32	76.2	75	65.8
NA	12	16.7	10	23.3			2	11.1	4	9.5	16	14.0
**Specific underlying conditions****												
Preterm birth	11	15.3	6	14.0	3	27.3	2	11.1	3	7.1	14	12.3
Asthma	3	4.2	1	2.3	2	18.2			2	4.8	5	4.4
Congenital heart disease***	4	5.6	2	4.7	1	9.1	1	5.6	1	2.4	5	4.4
Bronchopulmonary dysplasia	2	2.8	1	2.3	1	9.1			1	2.4	3	2.6
Down syndrome	2	2.8	1	2.3	1	9.1			1	2.4	3	2.6
Hypotonia	1	1.4	1	2.3							1	0.9

*NA, data not available; **Some patients had more than one underlying condition; ***In one case the diagnosis was probable heart disease (non-confirmed)

Supplementary Table S3. HRSV lineage distribution in each continent and Mexico for sequences reported between 2021 and 2024.

Lineage	Africa	Asia	Europe	North America	Oceania	South America	Mexico
A.2	-	-	-	-	-	-	-
A.2.1	-	-	-	-	-	-	-
A.2.1.1	-	-	-	-	-	-	-
A.3	-	-	0.03%	0.1%	-	-	-
A.D	2.7%	6.1%	0.2%	0.3%	0.6%	1.2%	-
A.D.1	23.6%	4.2%	34.8%	28.6%	6.3%	41.9%	59.7%
A.D.1.1	3.2%	-	-	0.4%	5.7%	-	-
A.D.1.2	-	-	-	-	9.0%	-	-
A.D.1.3	-	-	0.1%	-	26.7%	-	-
A.D.1.4	-	-	2.8%	0.6%	1.5%	4.0%	-
A.D.2	-	3.2%	0.8%	1.2%	-	-	-
A.D.2.1	-	-	0.03%	0.7%	-	-	-
A.D.2.2	-	0.5%	0.03%	-	-	-	-
A.D.2.2.1	-	-	0.1%	-	-	-	-
A.D.3	11.4%	68.6%	12.3%	14.7%	3.4%	29.0%	15.3%
A.D.3.1	6.1%	6.4%	8.5%	1.0%	15.6%	0.4%	-
A.D.4	9.7%	-	1.0%	-	-	0.8%	-
A.D.4.1	8.9%	-	-	-	-	-	-
A.D.5	19.0%	8.6%	12.2%	5.0%	0.2%	8.5%	-
A.D.5.1	10.5%	-	1.6%	1.6%	2.9%	-	1.40%
A.D.5.2	4.9%	2.2%	25.5%	45.1%	4.3%	14.1%	23.6%
A.D.5.3	-	0.2%	0.03%	0.8%	24.1%	-	-
Total	100%	100%	100%	100%	100%	100%	100%
B.2	-	-	-	-	-	-	-
B.3	-	-	-	-	-	-	-
B.4	-	-	-	-	-	-	-
B.D	-	0.4%	-	-	-	-	-
B.D.1	-	-	-	-	-	-	-
B.D.4	-	-	-	-	-	-	-
B.D.4.1	1.8%	2.7%	4.4%	-	0.2%	-	-
B.D.4.1.1	73.1%	41.3%	10.3%	6.5%	8.5%	6.6%	-
B.D.E.1	23.4%	49.0%	80.9%	93.0%	25.5%	93.4%	100.0%
B.D.E.2	1.2%	6.2%	0.1%	0.3%	0.1%	-	-
B.D.E.3	-	-	4.2%	0.2%	0.1%	-	-
B.D.E.4	-	-	0.1%	-	65.5%	-	-
Total	99%	100%	100%	100%	100%	100%	100%

Supplementary Table S4. Lineage specific amino acid and other changes present in HRSV-A sequences from Mexico

Sequence ID	Lineage assigned by amino acid signatures [4]	Other shared changes	Lineage assigned by phylogenetic analysis
HRSV/A/MEX/14099/2022	A.D.1 L142S	G106E+Y280H	A.D.1
HRSV/A/MEX/14101/2022	A.D.1 L142S	G106E+Y280H	A.D.1
HRSV/A/MEX/14103/2022	A.D.1 L142S	G106E+Y280H	A.D.1
HRSV/A/MEX/24115/2022	A.D.1 L142S	G106E+Y280H	A.D.1
HRSV/A/MEX/24123/2022	A.D.1 L142S	G106E+Y280H	A.D.1
HRSV/A/MEX/24131/2022	A.D.1 L142S	G106E+Y280H	A.D.1
HRSV/A/MEX/06069/2022	**A.D.1** L142S+T320A	G106E	A.D.1
HRSV/A/MEX/06070/2022	**A.D.1** L142S+T320A	G106E	A.D.1
HRSV/A/MEX/09071/2021	**A.D.1** L142S+T320A	G106E	A.D.1
HRSV/A/MEX/14086/2021	**A.D.1** L142S+T320A	G106E	A.D.1
HRSV/A/MEX/14104/2022	**A.D.1** L142S+T320A	G106E+Y280H	A.D.1
HRSV/A/MEX/14105/2022	**A.D.1** L142S+T320A	G106E+Y280H	A.D.1
HRSV/A/MEX/24109/2022	**A.D.1** L142S+T320A	A57T+S100N+ G224V+ S294P	A.D.1
HRSV/A/MEX/24110/2022	**A.D.1** L142S+T320A	A57T+S100N+ G224V+ S294P	A.D.1
HRSV/A/MEX/24111/2022	**A.D.1** L142S+T320A	A57T+S100N+ G224V+ S294P	A.D.1
HRSV/A/MEX/24113/2022	**A.D.1** L142S+T320A	A57T+S100N+ G224V+ S294P	A.D.1

HRSV/A/MEX/24116/2022	**A.D.1** L142S+T320A	A57T+S100N+ G224V+ S294P	A.D.1
HRSV/A/MEX/24117/2022	**A.D.1** L142S+T320A	A57T+S100N+ G224V+ S294P	A.D.1
HRSV/A/MEX/24120/2022	**A.D.1** L142S+T320A	A57T+S100N+ G224V+ S294P	A.D.1
HRSV/A/MEX/24122/2022	**A.D.1** L142S+T320A	A57T+S100N+ G224V+ S294P	A.D.1
HRSV/A/MEX/24125/2022	**A.D.1** L142S+T320A	A57T+S100N+ G224V+ S294P	A.D.1
HRSV/A/MEX/24126/2022	**A.D.1** L142S+T320A	A57T+S100N+ G224V+ S294P	A.D.1
HRSV/A/MEX/24128/2022	**A.D.1** L142S+T320A	A57T+S100N+ G224V+ S294P	A.D.1
HRSV/A/MEX/24129/2022	**A.D.1** L142S+T320A	A57T+S100N+ G224V+ S294P	A.D.1
HRSV/A/MEX/24132/2023	**A.D.1** L142S+T320A	A57T+S100N+ G224V+ S294P	A.D.1
HRSV/A/MEX/24133/2023	**A.D.1** L142S+T320A	A57T+S100N+ G224V+ S294P	A.D.1
HRSV/A/MEX/24134/2023	**A.D.1** L142S+T320A	A57T+S100N+ G224V+ S294P	A.D.1
HRSV/A/MEX/24135/2023	**A.D.1** L142S+T320A	A57T+S100N+ G224V+ S294P	A.D.1
HRSV/A/MEX/24137/2023	**A.D.1** L142S+T320A	A57T+S100N+ G224V+ S294P	A.D.1
HRSV/A/MEX/24138/2023	**A.D.1** L142S+T320A	A57T+S100N+ G224V+ S294P	A.D.1
HRSV/A/MEX/24162/2023	**A.D.1** L142S+T320A	A57T+S100N+ G224V+ S294P	A.D.1
HRSV/A/MEX/24166/2023	**A.D.1** L142S+T320A	A57T+S100N+ G224V+ S294P	A.D.1
HRSV/A/MEX/24171/2023	**A.D.1** L142S+T320A	A57T+S100N+ G224V+ S294P	A.D.1
HRSV/A/MEX/24177/2024	**A.D.1** L142S+T320A	A57T+S100N+ G224V+ S294P	A.D.1
HRSV/A/MEX/24180/2024	**A.D.1** L142S+T320A	A57T+S100N+ G224V+ S294P	A.D.1
HRSV/A/MEX/14092/2021	**A.D.1** L142S+T320A	G224V	A.D.1
HRSV/A/MEX/24139/2023	**A.D.1** L142S+T320A	G224V	A.D.1
HRSV/A/MEX/24145/2023	**A.D.1** L142S+T320A	G224V	A.D.1
HRSV/A/MEX/24150/2023	**A.D.1** L142S+T320A	G224V	A.D.1
HRSV/A/MEX/24154/2023	**A.D.1** L142S+T320A	G224V	A.D.1
HRSV/A/MEX/24161/2023	**A.D.1** L142S+T320A	G224V	A.D.1
HRSV/A/MEX/24165/2023	**A.D.1** L142S+T320A	G224V	A.D.1
HRSV/A/MEX/24167/2023	**A.D.1** L142S+T320A	G224V	A.D.1
HRSV/A/MEX/09080/2022	**A.D.3** T113I+V131D+N178G+ H258Q+ H266L	T245A+P298L	A.D.3
HRSV/A/MEX/14084/2021	**A.D.3** T113I+V131D+N178G+ H258Q+ H266L	K204N+P234S+N255D +E271K+P274L+P298L+ E308K	A.D.3

HRSV/A/MEX/14094/2021	**A.D.3** T113I+V131D+N178G+ H258Q+ H266L	K204N+P234S+N255D +E271K+P274L+P298L + E308K	A.D.3
HRSV/A/MEX/24114/2022	**A.D.3** T113I+V131D+N178G+ H258Q+ H266L	P274L+P298L	A.D.3
HRSV/A/MEX/24127/2022	**A.D.3** T113I+V131D+N178G+ H258Q+ H266L	K204R+T245A+P298L	A.D.3
HRSV/A/MEX/24141/2023	**A.D.3** T113I+V131D+N178G+ H258Q+ H266L	P146S+K204R+T245A+ P298L+S299G	A.D.3
HRSV/A/MEX/24143/2023	**A.D.3** T113I+V131D+N178G+ H258Q+ H266L	P146S+K204R+T245A+ P298L+S299G	A.D.3
HRSV/A/MEX/24151/2023	**A.D.3** T113I+V131D+N178G+ H258Q+ H266L	P146S+K204R+T245A+ P298L+S299G	A.D.3
HRSV/A/MEX/24168/2023	**A.D.3** T113I+V131D+N178G+ H258Q+ H266L	P146S+K204R+T245A+ P298L+S299G	A.D.3
HRSV/A/MEX/24170/2023	**A.D.3** T113I+V131D+N178G+ H258Q+ H266L	P146S+K204R+T245A+ P298L+S299G	A.D.3
HRSV/A/MEX/24175/2024	**A.D.3** T113I+V131D+N178G+ H258Q+ H266L	P146S+K204R+T245A+ P298L+S299G	A.D.3
HRSV/A/MEX/24159/2023	**A.D.5.1** A57V+T118I+ R209K+ T319I	L248I+S294P	A.D.5.1
HRSV/A/MEX/06067/2022	**A.D.5.2** A57V+ K209R	L248I+S270L+L310P	A.D.5.2
HRSV/A/MEX/14102/2022	**A.D.5.2** A57V+ K209R	L248I+S270L+L310P	A.D.5.2
HRSV/A/MEX/09072/2022	**A.D.5.2** A57V+ K209R	T235I+L248I+ L310P	A.D.5.2
HRSV/A/MEX/09076/2022	**A.D.5.2** A57V+ K209R	T235I+L248I+ L310P	A.D.5.2
HRSV/A/MEX/24121/2022	**A.D.5.2** A57V+ K209R	T235I+L248I+ L310P	A.D.5.2
HRSV/A/MEX/24130/2022	**A.D.5.2** A57V+ K209R	T235I+L248I+ L310P	A.D.5.2
HRSV/A/MEX/24144/2023	**A.D.5.2** A57V+ K209R	T235I+L248I	A.D.5.2
HRSV/A/MEX/24146/2023	**A.D.5.2** A57V+ K209R	T235I+L248I+ L310P	A.D.5.2
HRSV/A/MEX/24148/2023	**A.D.5.2** A57V+ K209R	T235I+L248I	A.D.5.2
HRSV/A/MEX/24149/2023	**A.D.5.2** A57V+ K209R	T235I+L248I	A.D.5.2
HRSV/A/MEX/24153/2023	**A.D.5.2** A57V+ K209R	T235I+L248I+ L310P	A.D.5.2
HRSV/A/MEX/24155/2023	**A.D.5.2** A57V+ K209R	T235I+L248I+ L310P	A.D.5.2
HRSV/A/MEX/24156/2023	**A.D.5.2** A57V+ K209R	T235I+L248I+ L310P	A.D.5.2
HRSV/A/MEX/24158/2023	**A.D.5.2** A57V+ K209R	T235I+L248I+ L310P	A.D.5.2
HRSV/A/MEX/24160/2023	**A.D.5.2** A57V+ K209R	T235I+L248I+ L310P	A.D.5.2
HRSV/A/MEX/24164/2023	**A.D.5.2** A57V+ K209R	T235I+L248I+ L310P	A.D.5.2
HRSV/A/MEX/24169/2023	**A.D.5.2** A57V+ K209R	T235I+L248I+ L310P	A.D.5.2

Supplementary Table S5. Lineage specific amino acid and other changes present in HRSV-B sequences from Mexico.

Sequence ID	Lineage assigned by amino acid signatures [4]	Other shared changes	Lineage assigned by phylogenetic analysis
HRSV/B/MEX/24118/2022	**B.D.E.1** S100G+P214S+ P221L+I252T+K256N+I268T+ S275P+Y285H	P109S	B.D.E.1

HRSV/B/MEX/09078/2022	**B.D.E.1** S100G+P214**F**+ P221L+I252T+K256N+I268T+ S275P+Y285H	P109S+ T139I+S265P	B.D.E.1
HRSV/B/MEX/09079/2023	**B.D.E.1** S100G+P214S+ P221L+I252T+K256N+I268T+ S275P+Y285H	P109S+ T139I+S265P	B.D.E.1
HRSV/B/MEX/09082/2023	**B.D.E.1** S100G+P214S+ P221L+I252T+K256N+I268T+ S275P+Y285H	P109S+ T139I+S265P	B.D.E.1
HRSV/B/MEX/09081/2023	**B.D.E.1** S100G+P214S+ P221L+I252T+K256N+I268T+ S275P+Y285H	P109S+ T139I+L217P S265P	B.D.E.1
HRSV/B/MEX/14083/2021	**B.D.E.1** S100G+P214S+ P221L+I252T+K256N+I268T+ S275P+Y285H	T139I+ S265P	B.D.E.1
HRSV/B/MEX/14085/2021	**B.D.E.1** S100G+P214S+ P221L+I252T+K256N+I268T+ S275P+Y285H	T139I+ S265P	B.D.E.1
HRSV/B/MEX/14088/2021	**B.D.E.1** S100G+P214S+ P221L+I252T+K256N+I268T+ S275P+Y285H	T139I+ S265P	B.D.E.1
HRSV/B/MEX/14090/2021	**B.D.E.1** S100G+P214S+ P221L+I252T+K256N+I268T+ S275P+Y285H	T139I+ S265P	B.D.E.1
HRSV/B/MEX/24124/2022	**B.D.E.1** S100G+P214S+ P221L+I252T+K256N+I268T+ S275P+Y285H	T139I+L217P S265P	B.D.E.1
HRSV/B/MEX/14087/2021	**B.D.E.1** S100G+P214S+ P221L+I252T+K256N+I268T+ S275P+Y285H	T139I	B.D.E.1
HRSV/B/MEX/14091/2021	**B.D.E.1** S100G+P214S+ P221L+I252T+K256N+I268T+ S275P+Y285H	T139I	B.D.E.1
HRSV/B/MEX/14093/2021	**B.D.E.1** S100G+P214S+ P221L+I252T+K256N+I268T+ S275P+Y285H	T139I	B.D.E.1
HRSV/B/MEX/14095/2021	**B.D.E.1** S100G+P214S+ P221L+I252T+K256N+I268T+ S275P+Y285H	T139I	B.D.E.1
HRSV/B/MEX/14096/2021	**B.D.E.1** S100G+P214S+ P221L+I252T+K256N+I268T+ S275P+Y285H	T139I	B.D.E.1
HRSV/B/MEX/09073/2022	**B.D.E.1** S100G+P214S+ P221L+I252T+K256N+I268T+ S275P+Y285H		B.D.E.1
HRSV/B/MEX/24107/2021	**B.D.E.1** S100G+P214S+ P221L+I252T+K256N+I268T+ S275P+Y285H		B.D.E.1
HRSV/B/MEX/14098/2022	**B.D.E.1** S100G+P214S+ P221L+I252T+K256N+I268T+ S275P+Y285H		B.D.E.1

HRSV/B/MEX/14100/2022	**B.D.E.1** S100G+P214S+ P221L+I252T+K256N+I268T+ S275P+Y285H		B.D.E.1
HRSV/B/MEX/24108/2021	**B.D.E.1** S100G+P214S+ P221L+I252T+K256N+I268T+ S275P+Y285H		B.D.E.1
HRSV/B/MEX/24112/2022	**B.D.E.1** S100G+P214S+ P221L+I252T+K256N+I268T+ S275P+Y285H		B.D.E.1
HRSV/B/MEX/24136/2023	**B.D.E.1** S100G+P214S+ P221L+I252T+K256N+I268T+ S275P+Y285H		B.D.E.1
HRSV/B/MEX/24147/2023	**B.D.E.1** S100G+P214S+ P221L+I252T+K256N+I268T+ S275P+Y285H		B.D.E.1
HRSV/B/MEX/24157/2023	**B.D.E.1** S100G+P214S+ P221L+I252T+K256N+I268T+ S275P+Y285H		B.D.E.1
HRSV/B/MEX/24173/2024	**B.D.E.1** S100G+P214S+ P221L+I252T+K256N+I268T+ S275P+Y285H		B.D.E.1
HRSV/B/MEX/24176/2024	**B.D.E.1** S100G+P214S+ P221L+I252T+K256N+I268T+ S275P+Y285H		B.D.E.1
HRSV/B/MEX/24178/2024	**B.D.E.1** S100G+P214S+ P221L+I252T+K256N+I268T+ S275P+Y285H		B.D.E.1
HRSV/B/MEX/09074/2022	**B.D.E.1** S100G+P214S+ P221L+I252T+K256N+I268T+ S275P+Y285H	L71P+ V76I	B.D.E.1
HRSV/B/MEX/09075/2022	**B.D.E.1** S100G+P214S+ P221L+I252T+K256N+I268T+ S275P+Y285H	L71P+ V76I	B.D.E.1
HRSV/B/MEX/09077/2022	**B.D.E.1** S100G+P214S+ P221L+I252T+K256N+I268T+ S275P+Y285H	L71P+V76I+L217P	B.D.E.1
HRSV/B/MEX/14089/2021	**B.D.E.1** S100G+P214S+ P221L+I252T+K256N+I268T+ S275P+Y285H	A42V	B.D.E.1
HRSV/B/MEX/14097/2021	**B.D.E.1** S100G+P214S+ P221L+I252T+K256N+I268T+ S275P+Y285H	A42V	B.D.E.1
HRSV/B/MEX/24119/2022	**B.D.E.1** P214S+ P221L+I252T+ I268T+S275P+Y285H	A42V+P109S+ T139I+ S265P	B.D.E.1
HRSV/B/MEX/16106/2022	**B.D.E.1** S100G+P214S+ P221L+I252T +I268T+S275P+Y285H		B.D.E.1
HRSV/B/MEX/24179/2024	**B.D.E.1** P214S+P221L+I252T+K256N +I268T+ S275P+Y285H		B.D.E.1
HRSV/B/MEX/06068/2022	**B.D.E.1** S100G+P214S+ P221L+I252T+K256N+I268T+Y285H		B.D.E.1
HRSV/B/MEX/24163/2023	**B.D.E.1** S100G+P214S+P221L+I252T +K256N+I268T+ S275P+Y285H	K85E	B.D.E.1

HRSV/B/MEX/24140/2023	**B.D.E.1** S100G+P214S+P221L+I268**V** **B.D.4.1.1** A131T+ T137I	K85E+P205S+ P229S+S243I	B.D.E.1
HRSV/B/MEX/24142/2023	**B.D.E.1** S100G+P214S+P221L+I268**V** **B.D.4.1.1** A131T+ T137I	K85E+P205S+ P229S+S243I	B.D.E.1
HRSV/B/MEX/24152/2023	**B.D.E.1** S100G+P214S+P221L+I268**V** **B.D.4.1.1** A131T+ T137I	K85E+P205S+ P229S+S243I	B.D.E.1
HRSV/B/MEX/24172/2024	**B.D.E.1** S100G+P214S+P221L+I268**V** **B.D.4.1.1** A131T+ T137I	K85E+P205S+ P229S+S243I	B.D.E.1
HRSV/B/MEX/24174/2024	**B.D.E.1** S100G+P214S+P221L+I268**V** **B.D.4.1.1** A131T+ T137I	K85E+P205S+ P229S+S243I	B.D.E.1

Letters in red color indicate a different amino acid from the parental and not reported by Goya et al. [4]

1. Zou, L.; Yi, L.; Wu, J.; Song, Y.C.; Huang, G.F.; Zhang, X.; Liang, L.; Ni, H.Z.; Pybus, O.G.; Ke, C.W.; et al. Evolution and Transmission of Respiratory Syncytial Group A (RSV-A) Viruses in Guangdong, China 2008-2015. *Front Microbiol*. 2016, 7, https://doi.org/10.3389/fmicb.2016.01263
2. M, Höneman.; S, Thiem.; S, Bergs.; T, Berthold.; C, Propach.; M, Siekmeyer.; A, Frille.; T, Wallborn.; M, Maier.; C, Pietsch. In-Depth Analysis of the Re-Emergence of Respiratory Syncytial Virus at a Tertiary Care Hospital in Germany in the Summer of 2021 after the Alleviation of Non-Pharmaceutical Interventions Due to the SARS-CoV-2 Pandemic. Viruses. 2023, 15. https://doi.org/10.3390/v15040877
3. MA, L.A.; M, P.M.; E, L.M.; MS, M.T.; B, P.P. Transmission of Respiratory Syncytial Virus genotypes in Cali, Colombia. *Influenza and Other Respiratory Viruses*. 2021, 15, 521-528. https://doi.org/10.1111/irv.12833
4. Goya, S.; Ruis, C.; Neher, R.A.; Meijer, A.; Aziz, A.; Hinrichs, A.S.; von Gottberg, A.; Roemer, C.; Amoako, D.G.; Acuna, D.; et al. Standardized phylogenetic classification of human respiratory syncytial virus below the subgroup level. Emerg. Infect. Dis. 2024, 30, 1631–1641
